# Supplementary material for: The Dual Prey-Inactivation Strategy of Spiders—In-Depth Venomic Analysis of Cupiennius salei
Source: Toxins (Basel). 2019 Mar 19;11(3):167. doi: 10.3390/toxins11030167 (PMC6468893; doi:10.3390/toxins11030167)
Supplement: Supplementary file 1 [file toxins-11-00167-s001.zip › Supplementary Fig S1.1-S1.19/Supplementary Fig S1.18.pdf]

Supplementary Fig S1.18

CLUSTAL O(1.2.4) multiple sequence alignment: Tachylectin 5A-like proteins

```
TL5A_1_CUPSA                  ----MHLLLVLFIVSSAI-SSEAGESGKCDSEWSQNVTEYSRDSFIVSLLNDAERLLAKA
TL5A_2_CUPSA                  ----MHLLLVLFIVSSAI-SSEAGESGKCDSEWSQNVTEYSRDSFIVSLLNDAERLLAKA
TL5A_ALOMA                    -------VLLCFTCMCIFSYSSSAKEEKCTSFLSNNITEQTQSSIIVTLLNNAKNILEAA
TL5A_VIRFA                    ----VKMYIVLIICISVV-SSEATNDGKCSSWLFQNITEYNRASFITNLLSRAVNLLEDA
tr_A0A087T8T0_STEMI           MNLRMRLYFIVLNAILVACTAC------------KLKDSIDASSIVVDLLNSATVLLNDA
gi_XP_015905659.1_PARTE       --MKSLLVILCFILFLSNCDGKGKSSSACS----K---E-EK---TVSYLDMAMEMIDKA
                                                          . :   :        .  .  *. *  ::  *

TL5A_1_CUPSA                  KRHASAGIGRAVATPSCEKPMDCFEIHEKDQGAKSGEYKIWPRSRISN-AGIVVYCDMDD
TL5A_2_CUPSA                  KRHASTGIGRAVATPSCEKPMDCFEIHEKDQGAKSGEYKIWPRSRISN-AGIVVYCDMDD
TL5A_ALOMA                    QKYATVKPRCSVVGTTCDKPMDCAEVKEKNAFAYSSVYKIWPRNRVVS-GPVEVYCDMED
TL5A_VIRFA                    KKYASMRTSCTNSQPMCEKPKDCFDIYEKNPTATSGVHKVWPSSRISS-DAIRVYCDMDD
tr_A0A087T8T0_STEMI           KKYANKCSSCTDAYLTLNKPMDCAEIKMNSKTSKSGIYDIWPANRIIK-TSIKVYCDMED
gi_XP_015905659.1_PARTE       KEN-YISSQNSKFQALTSKPLDCEELL-KSGTNKSGVYVIWPQNRIMDRKPLDVYCDMET
                              :.        :      .** ** ::  :.    *. : :** .*: .   : *****:

TL5A_1_CUPSA                  DGGSWTVIQRRGDFNSGPDYFYRDWAAYKAGFGDPMKDFWLGNDNIFALTNQRANSVKFI
TL5A_2_CUPSA                  DGGSWTVIQRRGDFNSGPDYFYRDWAAYKAGFGDPMKDFWLGNDNIFALTNQRANSVKFI
TL5A_ALOMA                    DGGAWTVLQRRGDFDSAKDYFYKDWQSYKKGFGDPAKDFWLGNDNIFALTNQRSNIVKFA
TL5A_VIRFA                    DGGAWTVIQRRGDFDSGKNYFFREWEEYKKGFGSLEKDFWLGNDNIFALTNQRSNSIKFT
tr_A0A087T8T0_STEMI           DNGGWTVMQRRGDFGSAKDYFYRDWVSYKNGFGDLMKDFWLGNDNTFALTNERSNQIKIT
gi_XP_015905659.1_PARTE       DGGGWTVIQRRGNFSMPKDYFFREWLSYRTGFGDVEKEFWLGNDNIFALTNQRLYSVRFD
                              *.*.***:****:*.   :**:::*  *: ***.  *:******* *****:*   :::

TL5A_1_CUPSA                  LSDWEANTTYAVYDEFWIDDEINKYTLHVKGYRGTAGDSFVG-HNGAPFSTKDKDNDRYD
TL5A_2_CUPSA                  LSDWEANTTYAVYDEFWIDDEINKYTLHVKGYRGTAGDSFVG-HNGAPFSTKDKDNDRYD
TL5A_ALOMA                    LADWEKNQTYATYDEFWIDDESHKYTMHVSGYRGTAGDSFSD-TNGLPFTTKDQDNDKHD
TL5A_VIRFA                    LTDWEGNTTYAIYDEFWIDDEFNKYTAHVKGYRGTAGDSFSG-TNEAPFSTKDRDNDIYD
tr_A0A087T8T0_STEMI           LTDWEGNKTYALYDEFWIDDEYHKYTAHVMGYRGTAGDSFSG-VNGVPFSTKDKDNDTHE
gi_XP_015905659.1_PARTE       MKDMAGESRYALYDRFWIDDEDHLYALHISEYSGNAGDSMVNVHNNMKFSTKDKDNDSHG
                              : *   :  ** **.****** : *: *:  * *.****: .  *   *:***:*** :

TL5A_1_CUPSA                  KNCAELFKGGWWYTACHASNLNGLYLKGPHKSHADGVEWQLFRGHYYSLKDTVIKIRPRD
TL5A_2_CUPSA                  KNCAELFKGGWWYTACHASNLNGLYLKGPHKSHADGVEWQLFRGHYYSLKDTVIKIRPRD
TL5A_ALOMA                    KNCAVQFKGAWWYTACHSANLNGMYLKGAHESHADGVEWHGFRGHYYSLKDTVIKIRPRE
TL5A_VIRFA                    KNCADIFKGAWWYRACHNSNLNGLYLKGVHDSHADGVEWQSFRGHKYSLKDTVIKLRPKA
tr_A0A087T8T0_STEMI           GSCAVSYKGAWWYTKCHSSNLNGLYLKGPHESHADGVEWHGFRGHYYSLKDTVMKVRSID
gi_XP_015905659.1_PARTE       DNCAQMFKGGWWYGSCHESNLNGLNLRGKHDTHADGVNWKSFRGHNESLQNTEIKIRPKN
                               .**   :**.***  ** :****: *:* *.:*****:*: ****  **::* :*:*

TL5A_1_CUPSA                  FKGATQDA---T-PQ
TL5A_2_CUPSA                  FKGATQDA---T-PQ
TL5A_ALOMA                    FQKKPDGV---T-P-
TL5A_VIRFA                    FKATRMDV---T-PE
tr_A0A087T8T0_STEMI           YLTTEKDRTDST-PQ
gi_XP_015905659.1_PARTE       FKNNPSPIEEYETPK
                              :            *
```

Percent Identity Matrix - created by Clustal2.1

1: TL5A_1_CUPSA	100.00	99.67	62.33	67.76	59.73	51.89
2: TL5A_2_CUPSA	99.67	100.00	62.33	67.76	59.73	51.89
3: TL5A_ALOMA	62.33	62.33	100.00	61.67	61.59	49.65
4: TL5A_VIRFA	67.76	67.76	61.67	100.00	59.39	49.48
5: tr_A0A087T8T0_STEMI	59.73	59.73	61.59	59.39	100.00	47.20
6: gi_XP_015905659.1_PARTE	51.89	51.89	49.65	49.48	47.20	100.00

Proteomic coverage trypsin

>TL5A_1_CUPSA tachylectin 5A-like isoform 1

MHLLLVLFIV SSAISSEA**GE SGKCDSEWSQ NVTEYSRDSF IVSLLNDAER LLAK**AKRHAS **A**GIGR**AVATP SCEKPMDCFE**
IHEKDQGAKS GEYKIWPRSR ISNAGIVVYC DMDDDGGSWT VIQRRGDFNS GPDYFYRDWA AYK**AGFGDPM KDFWLGNDNI**
FALTNQRANS VKFILSDWEA NTTYAVYDEF WIDDEINKYT LHVK**GYRGTA GDSFVGHNGA PFSTK**DKDND R**YDKNCAELF**
KGGWWYTACH ASNLNGLYLK GPHK**SHADGV EWQLFRGHYY SLKDTVIK**IR PR**DFKGATQD ATPQ**

>TL5A_2_CUPSA tachylectin 5A-like isoform 2

MHLLLVLFIV SSAISSEA**GE SGKCDSEWSQ NVTEYSRDSF IVSLLNDAER LLAK**AKRHAS **T**GIGR**AVATP SCEKPMDCFE**
IHEKDQGAKS GEYKIWPRSR ISNAGIVVYC DMDDDGGSWT VIQRRGDFNS GPDYFYRDWA AYK**AGFGDPM KDFWLGNDNI**
FALTNQRANS VKFILSDWEA NTTYAVYDEF WIDDEINKYT LHVK**GYRGTA GDSFVGHNGA PFSTK**DKDND R**YDKNCAELF**
KGGWWYTACH ASNLNGLYLK GPHK**SHADGV EWQLFRGHYY SLKDTVIK**IR PR**DFKGATQD ATPQ**

Proteomic coverage chymotrypsin

>TL5A_1_CUPSA tachylectin 5A-like isoform 1

MHLLLVLFIV SSAISSEAGE SGKCDSEWSQ NVTEY**SRDSF IVSLLNDAER L**LAKAKRHAS **A**GIGR**AVATP SCEKPMDCFE**
IHEKDQGAKS GEYKIWPRSR ISNAGIVVYC DMDDDGGSWT VIQRRGDFNS GPDYFYRDWA AY**KAGFGDPM KDF**WLGNDNI
F**ALTNQRANS VKF**ILSDWEA NTTYAVY**DEF WIDDEINKYT LHVKGYRGTA GDSFVGHNGA PF**STKDKDND RYDKNCAELF
KGGWWY**TACH ASNLNGLY**LK GPHKSHADGV EWQLFRGHYY **SLKDTVIKIR PRDFKGATQD ATPQ**

>TL5A_2_CUPSA tachylectin 5A-like isoform 2

MHLLLVLFIV SSAISSEAGE SGKCDSEWSQ NVTEY**SRDSF IVSLLNDAER L**LAKAKRHAS **T**GIGR**AVATP SCEKPMDCFE**
IHEKDQGAKS GEYKIWPRSR ISNAGIVVYC DMDDDGGSWT VIQRRGDFNS GPDYFYRDWA AY**KAGFGDPM KDF**WLGNDNI
F**ALTNQRANS VKF**ILSDWEA NTTYAVY**DEF WIDDEINKYT LHVKGYRGTA GDSFVGHNGA PF**STKDKDND RYDKNCAELF
KGGWWY**TACH ASNLNGLY**LK GPHKSHADGV EWQLFRGHYY **SLKDTVIKIR PRDFKGATQD ATPQ**

Supplementary Fig S1.18 Multiple sequence alignment of TL5A from *C. salei* (TL5A_1_CUPSA [MH766646], TL5A_2_CUPSA [MH766647]), *A. marikovskyi* (TL5A_ALOMA), *V. fasciatus* (TL5A_VIRFA), *S. mimosarum* (tr_A0A087T8T0_STEMI), and *P. tepidariorum* (gi_XP_015905659.1_PARTE). Signal peptides are marked in black characters, cysteines in white with a black box, positively charged amino acids in pink, negatively charged amino acids in blue, hydrophobic amino acids in red and polar amino acids in green. A possible polymerization pocket is highlighted in yellow and a Ca^{2+} binding site in gray.
